# Supplementary material for: Sipa1 deficiency unleashes a host-immune mechanism eradicating chronic myelogenous leukemia-initiating cells
Source: Nat Commun. 2018 Mar 2;9:914. doi: 10.1038/s41467-018-03307-8 (PMC5834470; doi:10.1038/s41467-018-03307-8)
Supplement: Supplementary file 1 — Supplementary Information [file 41467_2018_3307_MOESM1_ESM.pdf]

Supplementary Information

A potent immune mechanism eradicating chronic myelogenous leukemia–initiating cells uncovered by *Sipa1* deficiency

Xu et al.

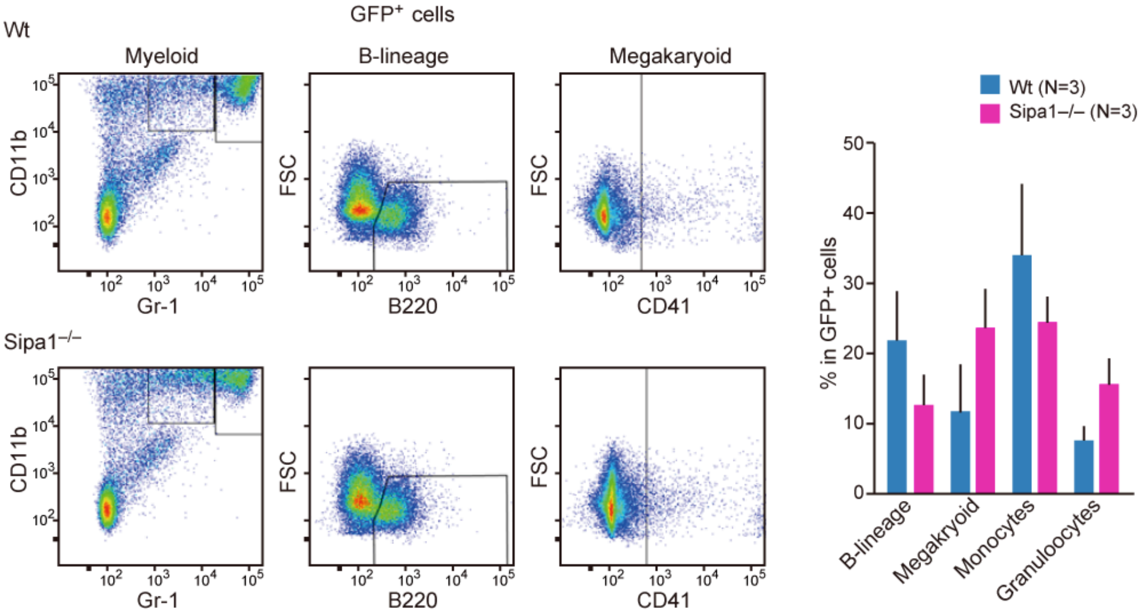

Supplementary Figure 1. *Bcr-Abl*$^{+}$ HPCs show comparable lineage differentiation profiles in Wt and *Sipa1*$^{-/-}$ mice. *Bcr-Abl*$^{+}$ HPCs were intravenously injected into Wt and *Sipa1*$^{-/-}$ mice, and 9 days later the BM cells were multi-color analyzed for indicated markers. Representative FACS profiles of myeloid, B-lineage, and megakaryocytes at the GFP^{+} cell gate are shown. The means and SEs of the proportions of indicated cell lineages in the GFP^{+} population of three mice are also indicated. Similar results were obtained in 3 experiments, and no statistically significant difference of the proportions of differentiated cell lineages was observed between Wt and Sipa1$^{-/-}$ mice.

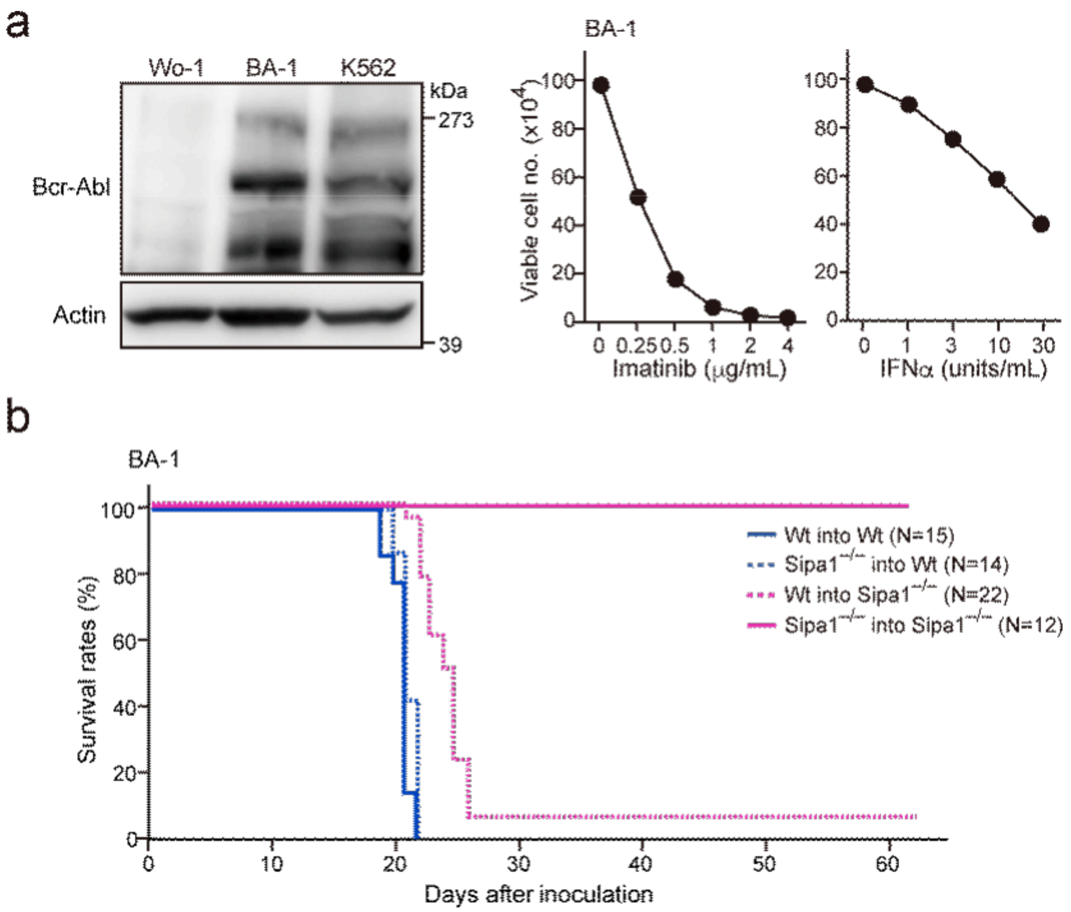

Supplementary Figure 2. *Sipa1*$^{-/-}$ mice show resistance to the *Bcr-Abl*$^{+}$ leukemia cell line, BA-1, which requires both hematopoietic and non-hematopoietic cell components. (**a**) The mouse BA-1 cell line expresses p210Bcr-Abl (Left). Mouse Wo-1 T-ALL and human K562 CML cell lines served as negative and positive controls, respectively. Uncropped images are shown in Supplementary Fig. 10. BA-1 cells were cultured in the presence of varying concentrations of imatinib or IFN-α for 4 days, and the viable cell numbers were counted (right). The means of triplicate cultures are indicated. (**b**) BA-1 cells were intravenously injected into the indicated numbers of BM chimeric mice between Wt and *Sipa1*$^{-/-}$ mice.

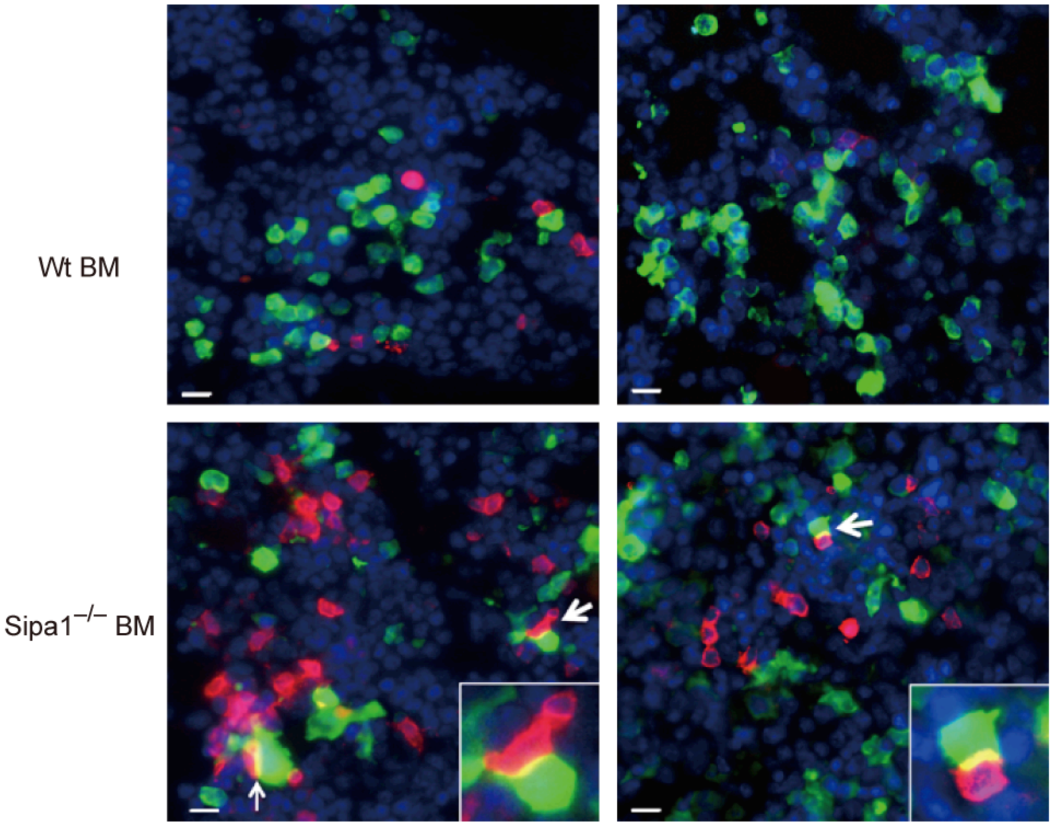

Supplementary Figure 3. *Bcr-Abl*$^{+}$ CML cells in *Sipa1*$^{-/-}$ BM are associated with greater numbers of T cells than those in Wt BM. *Bcr-Abl*$^{+}$ HPCs were intravenously injected into Wt and *Sipa1*$^{-/-}$mice, and 9 days later the femoral bones were sectioned, fixed, and two-color immunostained with anti-GFP (green) and anti-CD3 (red) antibodies. Arrows indicate the conjugates of CML and T cells. The enlarged images (thick arrows) are also shown. Scale bars; 10 μm. Similar results were obtained in three different samples.

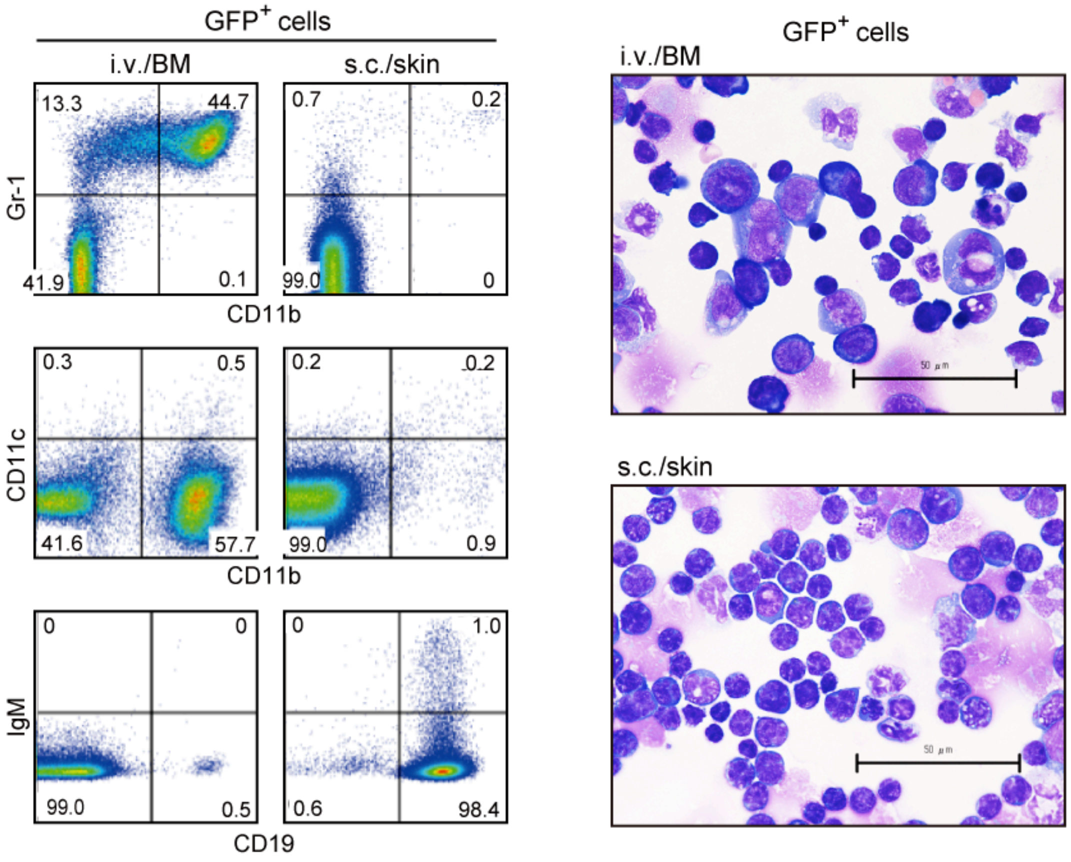

Supplementary Figure 4. *Bcr-Abl*$^+$ HPCs robustly proliferate in both BM and subcutaneous tissues of Wt mice with distinct lineage differentiation profiles. Primary *Bcr-Abl*$^+$ BM HPCs were injected intravenously (i.v.) or subcutaneously (s.c.) in Matrigel matrix in unirradiated Wt B6 mice. The GFP$^+$ cells in the BM and subcutaneous tumor, respectively, were FACS-analyzed for differentiation markers of myeloid lineages (CD11b, Gr-1), dendritic cells (CD11c), and B-lineage cells (CD19, IgM) (left). Giemsa-stained pictures confirmed the dominance of immature myeloid cells and erythroblasts in the BM and homogenous lymphoblastic cells in the subcutaneous tumor (right). Scale bars; 50μm. Essentially similar results were obtained in 5 mice.

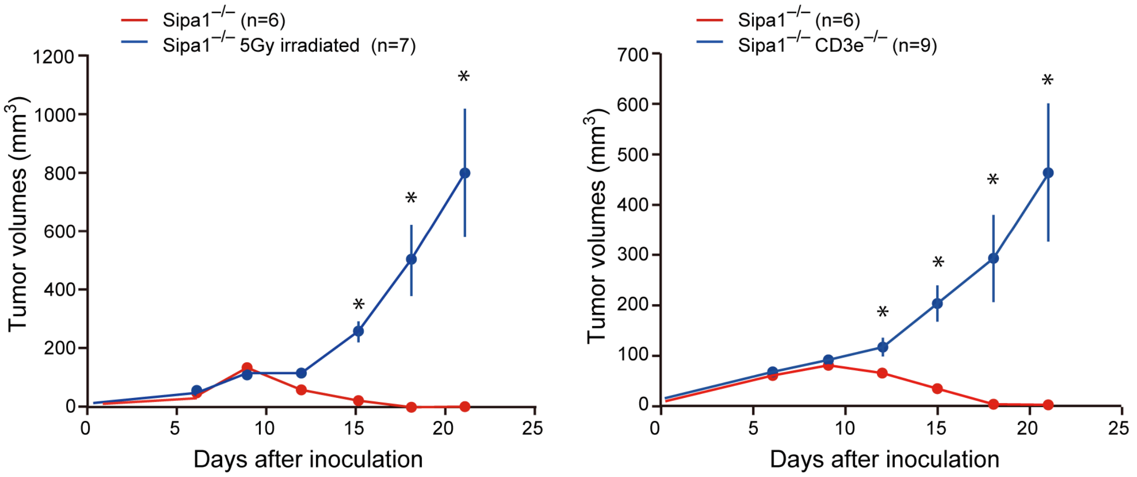

Supplementary Figure 5. Resistance of *Sipa1*$^{-/-}$ mice to subcutaneous *Bcr-Abl*$^{+}$ HPCs is also radiosensitive and dependent on T cells. *Bcr-Abl*$^{+}$ HPCs in Matrigel matrix were injected subcutaneously into *Sipa1*$^{-/-}$ mice that were untreated, 5Gy-irradiated (left), or at the *Cd3e*$^{-/-}$ genetic background (right). The means and SE of the tumor volumes of indicated numbers of mice are indicated. *; p<0.05 (two-tailed unpaired Student's t-test).

Enrichment plot (19,000 genes)

Mesenchymal lineage genes

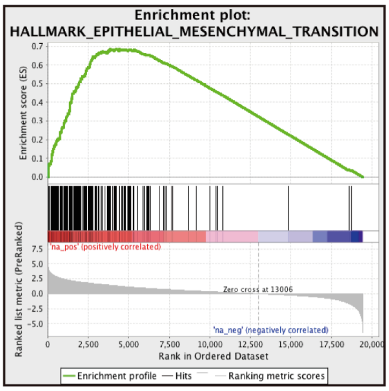

	Genes	Rank	Score		Genes	Rank	Score
1	MYLK	45	4.310	21	PRRX1	477	2.921
2	COL8A2	57	4.199	22	APLP1	533	2.855
3	IGFBP3	67	4.089	23	LOX	537	2.853
4	ABI3BP	69	4.077	24	TIMP3	563	2.836
5	POSTN	73	4.055	25	PTHLH	607	2.778
6	EDIL3	75	4.037	26	LRP1	693	2.700
7	GJA1	133	3.683	27	ENO2	757	2.643
8	COL5A2	220	3.392	28	COL6A2	761	2.641
9	COL1A2	221	3.389	29	THBS2	763	2.638
10	LAMA1	251	3.319	30	PVR	764	2.637
11	BGN	252	3.317	31	LAMA2	773	2.629
12	COL3A1	262	3.304	32	IL6	819	2.592
13	FN1	265	3.292	33	ADAM12	849	2.571
14	NID2	295	3.227	34	PLOD1	860	2.560
15	FBN2	301	3.215	35	SERPINE2	877	2.548
16	COL6A3	340	3.137	36	WNT5A	919	2.517
17	CTHRC1	347	3.132	37	SLIT2	980	2.467
18	MATN2	398	3.048	38	SPP1	981	2.467
19	PLOD2	413	3.014	39	SGCD	989	2.464
20	CDH6	435	2.985	40	LRRC15	994	2.461

ECM/Receptor genes

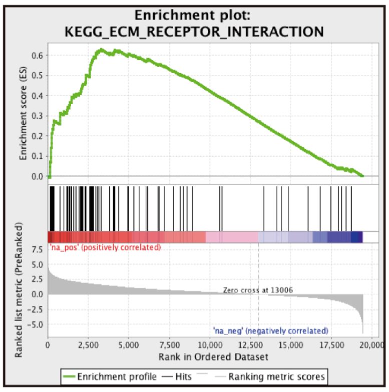

	Genes	Rank	Score		Genes	Rank	Score
1	ITGB8	146	3.630	21	SDC2	1461	2.177
2	ITGA1	147	3.630	22	COL5A3	1494	2.162
3	LAMB1	152	3.610	23	ITGB5	1592	2.114
4	ITGA8	190	3.488	24	COL6A1	1758	2.058
5	COL5A2	220	3.392	25	LAMB3	1961	1.975
6	COL1A2	221	3.389	26	HSPG2	2036	1.951
7	LAMA1	251	3.319	27	COL4A4	2094	1.933
8	COL3A1	262	3.304	28	SDC3	2113	1.929
9	FN1	265	3.292	29	COL6A6	2176	1.907
10	LAMA4	283	3.255	30	TNXB	2313	1.859
11	COL6A3	340	3.137	31	LAMB2	2377	1.841
12	ITGA11	381	3.083	32	THBS1	2379	1.840
13	COL6A2	761	2.641	33	GP1BB	2383	1.839
14	THBS2	763	2.638	34	ITGA9	2580	1.784
15	LAMA2	773	2.629	35	COL2A1	2582	1.784
16	SPP1	981	2.467	36	COL5A1	2584	1.783
17	SDC1	1181	2.333	37	CD44	2626	1.768
18	TNC	1217	2.306	38	ITGB1	2662	1.756
19	ITGA7	1347	2.233	39	LAMC2	2710	1.740
20	COMP	1385	2.214	40	LAMC1	2744	1.729

Collagen genes

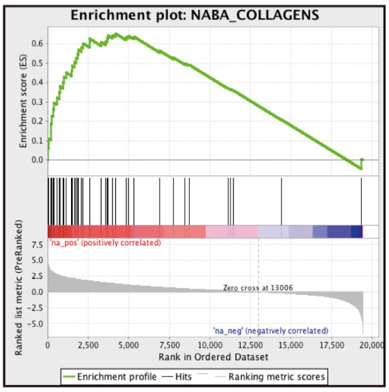

	Genes	Rank	Score		Genes	Rank	Score
1	COL6A5	18	4.838	21	COL6A6	2176	1.907
2	COL8A2	57	4.199	22	COL2A1	2582	1.784
3	COL5A2	220	3.392	23	COL5A1	2584	1.783
4	COL1A2	221	3.389	24	COL1A1	3300	1.569
5	COL3A1	262	3.304	25	COL28A1	3591	1.488
6	COL6A3	340	3.137	26	COL20A1	3675	1.464
7	COL8A1	402	3.039	27	COL18A1	3732	1.449
8	COL17A1	564	2.835	28	COL4A3	4001	1.381
9	COL23A1	735	2.659	29	COL7A1	4176	1.337
10	COL6A2	761	2.641				
11	COL24A1	951	2.487				
12	COL13A1	1016	2.444				
13	COL12A1	1135	2.352				
14	COL5A3	1494	2.162				
15	COL16A1	1528	2.149				
16	COL15A1	1644	2.097				
17	COL6A1	1758	2.058				
18	COL4A5	1853	2.019				
19	COL14A1	1912	1.993				
20	COL4A4	2094	1.933				

FGF/Receptor genes

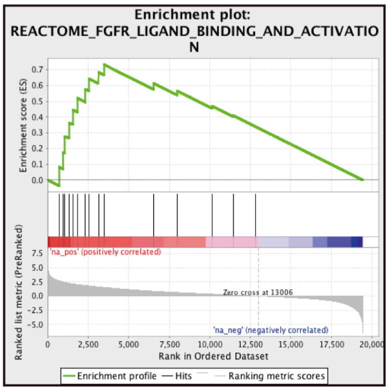

1	FGF18	701	2.687
2	FGFR1	971	2.474
3	KLB	1059	2.410
4	FGF1	1310	2.252
5	FGF9	1558	2.131
6	FGF10	1833	2.029
7	FGF2	2315	1.859
8	FGFR4	2537	1.795
9	FGF7	3137	1.611
10	FGF23	3475	1.521

Supplementary Figure 6. Intratumor MSCs of *Sipa1*$^{-/-}$ mice show enhanced expression of selected gene sets compared to those of Wt mice. MSCs (GFP^{-} CD45^{-}Ter119^{-} CD31^{-}) were sorted from the tumors after subcutaneous injection of *Bcr-Abl*$^{+}$ HPCs into Wt and *Sipa1*$^{-/-}$ mice, and the RNAs were subjected to microarray analysis. Gene set enrichment analysis indicated increased expression of the indicated gene sets in the MSCs of *Sipa1*$^{-/-}$ mice. The top 10–40 genes that increased in each gene set are listed. The data is available in the GEO repository, NCBI (GSE108002, https://www.ncbi.nlm.nih.gov/geo/query/acc.cgi?acc=GSE108002).

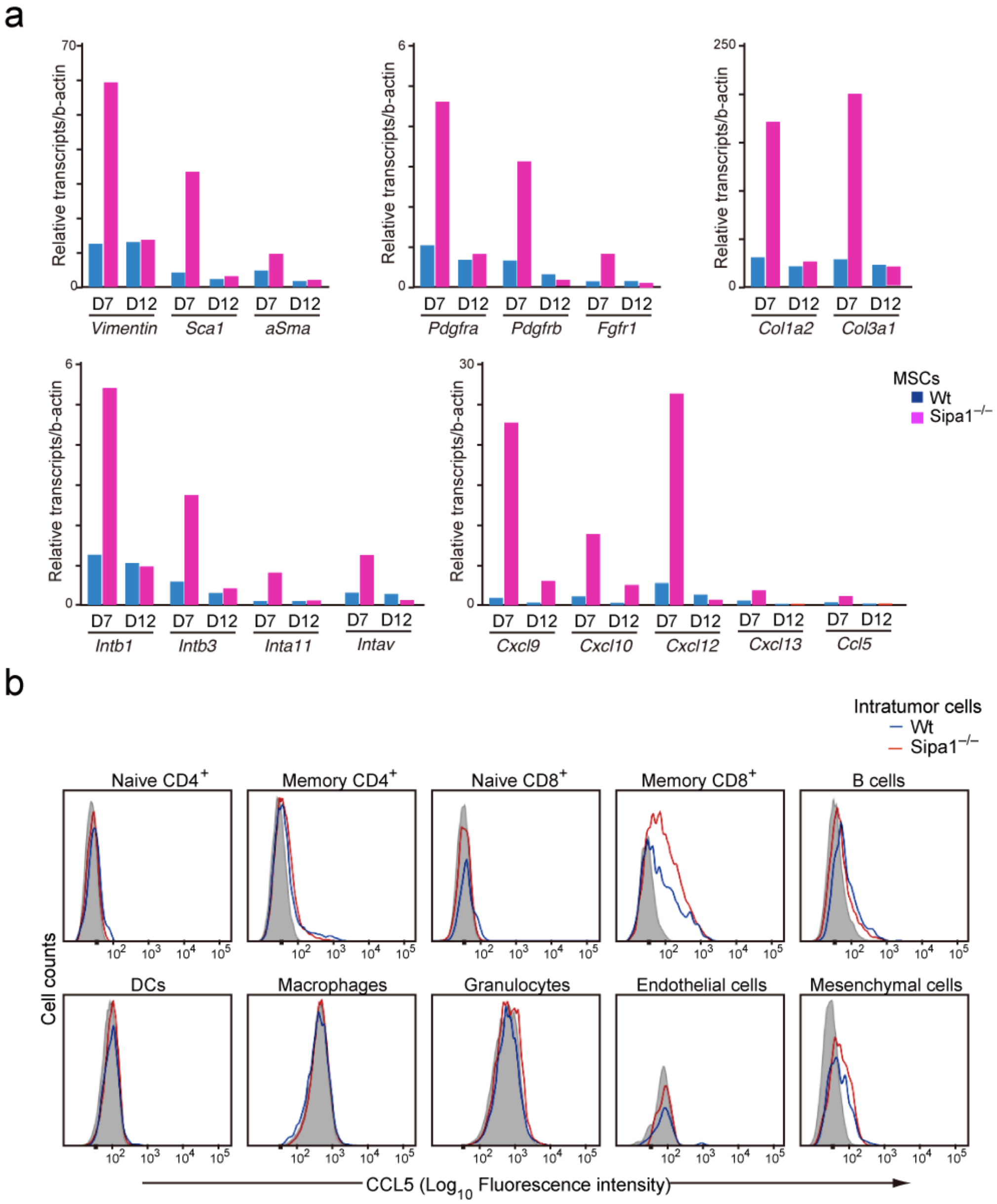

Supplementary Figure 7. MSCs are activated transiently during the early stage including *Cxcl9*, while Ccl5 is exclusively produced by memory CD8^{+} T cells at later stage in *Bcr-Abl*$^{+}$ tumor tissue of *Sipa1*$^{-/-}$ host. (**a**) *Bcr-Abl*$^{+}$ HPCs were subcutaneously injected in collagen matrix into Wt and *Sipa1*$^{-/-}$ mice, and the tumor tissues were resected on day 7 and day 12. MSCs (GFP^{-}CD45^{-}Ter119^{-}CD31^{-}) were sorted and subjected to quantitative PCR for the indicated genes. Contamination of GFP^{+} cells in the sorted cell population was less than 0.5%. Tumors of 3 mice were pooled for one experiment, and similar results were obtained in two independent experiments. (**b**) Subcutaneous *Bcr-Abl*$^{+}$ tumor tissues on day 12 were dispersed into a cell suspension in collagenase and DNase I solution, and the intracellular expression of Ccl5 was analyzed in the indicated cell populations. Tumors of 3 mice were pooled for one experiments, and similar results were obtained in two independent experiments.

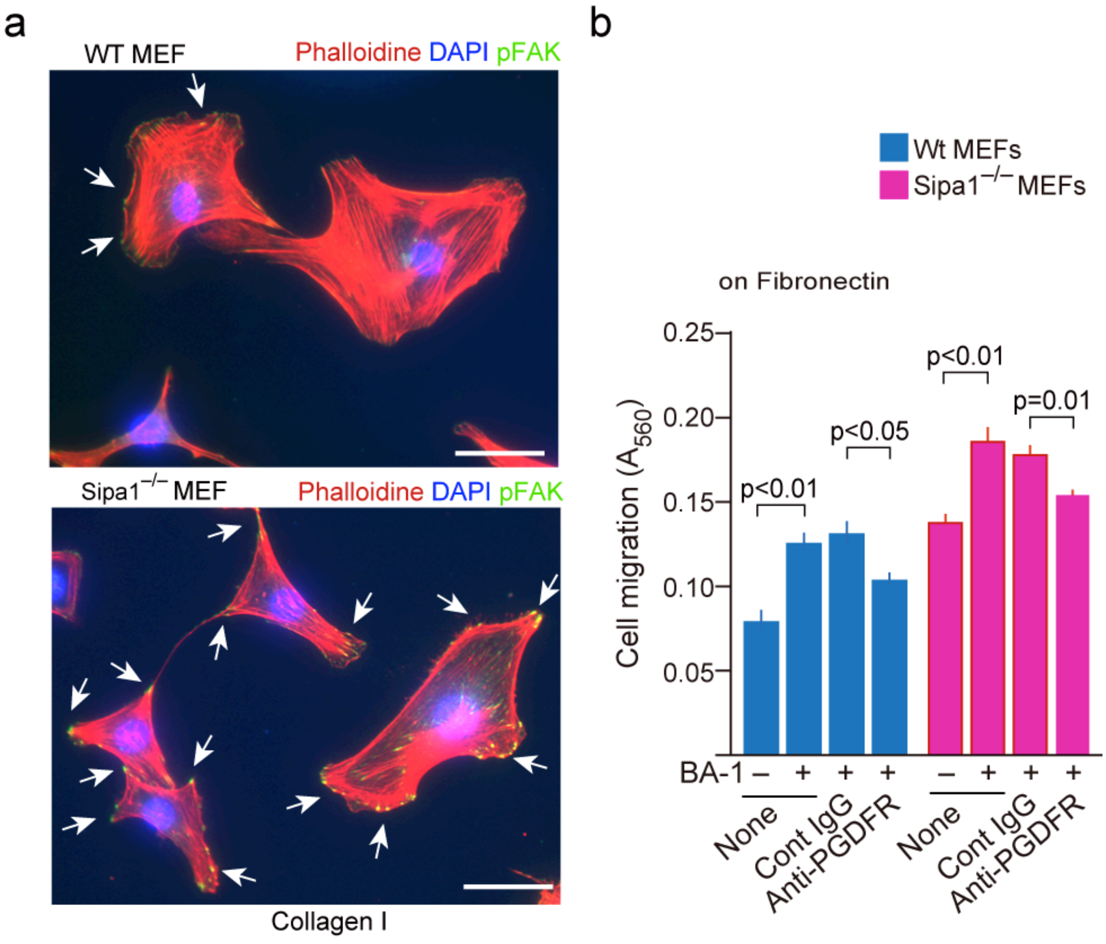

Supplementary Figure 8. *Sipa1*$^{-/-}$ MEFs show a markedly enhanced phosphorylation of FAK on collagen and directed migration to BA-1 cells that is inhibited in the presence of anti-PDGFR antibody. (**a**) Wt and *Sipa1*$^{-/-}$ MEFs were cultured on collagen I–coated cover slips in the medium containing 0.1% FCS and immunostained with anti-phospho-focal adhesion kinase (p-FAK). Arrows indicate p-FAK^{+} focal adhesion sites. Scale bars; 40 µm. (**b**) Directed migration of Wt and *Sipa1*$^{-/-}$ MEFs to BA-1 cells were assessed in the presence of fibronectin using the Boyden chamber. Anti-PDGFRα antibody or isotype-matched control IgG was included in the lower chambers with BA-1 cells. Means and SEs of quadruplicate culture are shown, and similar results were obtained in two independent experiments. *p* values; two-tailed unpaired Student's *t*-test.

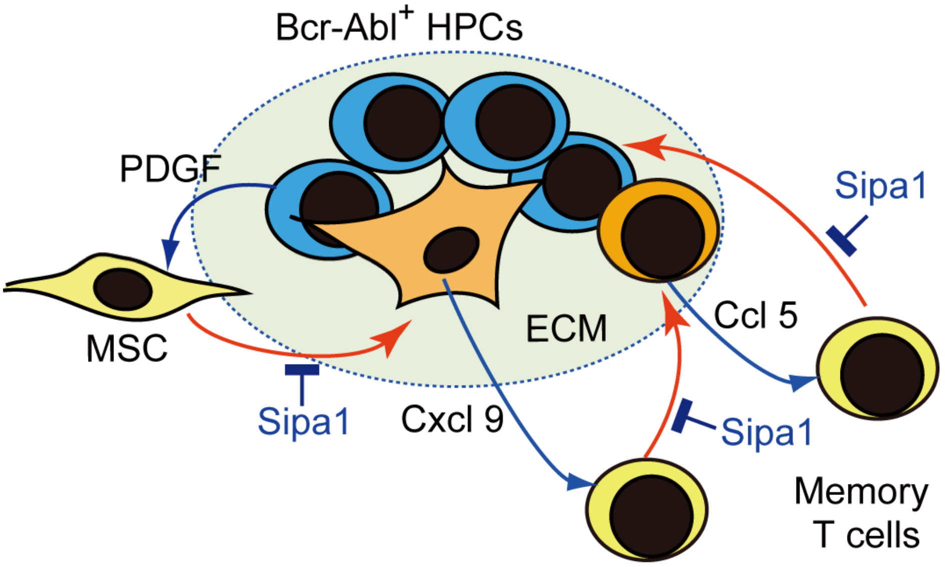

Supplementary Figure 9. Schematic representation of the interplay between MSCs and T cells in *Bcr-Abl*$^{+}$ tumor tissue of *Sipa1*$^{-/-}$ host in response to *Bcr-Abl*$^{+}$ CML cells. In the *Sipa1*$^{-/-}$ host, MSCs are strongly activated by *Bcr-Abl*$^{+}$ cells via PDGF, at least in part, and show enhanced directed migration to the CML cells. Concurrently, the MSCs are activated for gene sets including ECM and the related receptors and also secrete abundant Cxcl9 at the vicinity of CML cells, through which *Sipa1*$^{-/-}$ memory T cells that have enhanced chemotactic activity are efficiently recruited inside the tumor tissue. Such recruited T cells, in particular CD8^{+} cells, secrete Ccl5, which further amplifies the T cell recruitment to the tumor tissue and may also show potent TCR-costimulatory activity. We propose that such an endogenous cellular interplay in tumor tissue results in the spontaneous eradication of CML disease in *Sipa1*$^{-/-}$ host. Blue arrows indicate chemotactic effects, and red arrows show cell activation and migration. Blue "T" marks indicate the negative regulation by endogenous Sipa1.

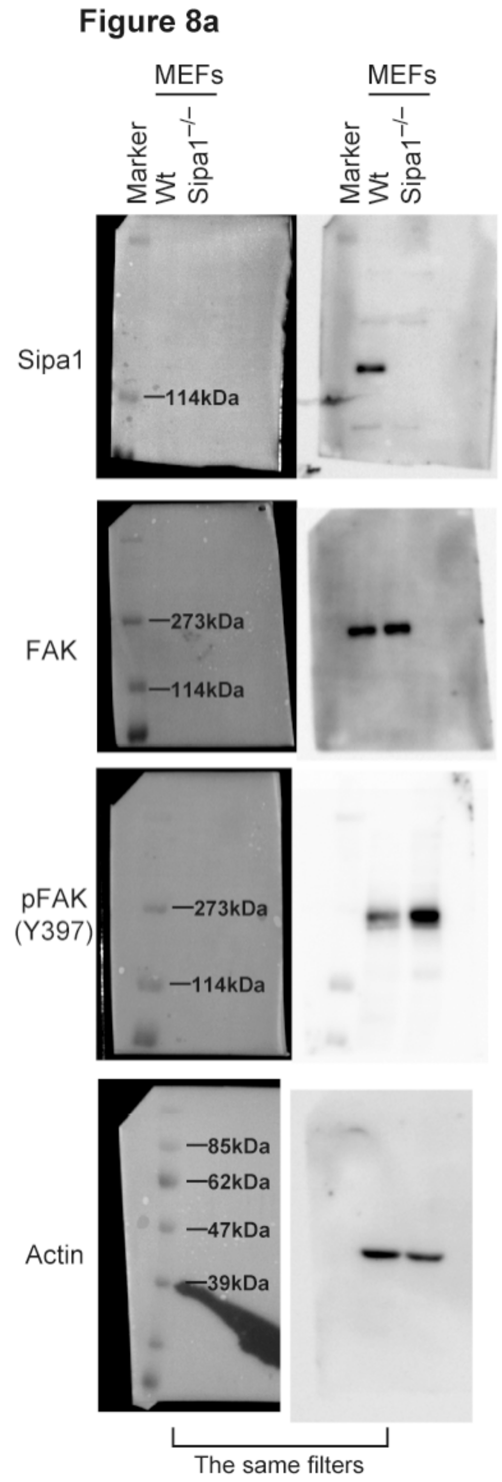

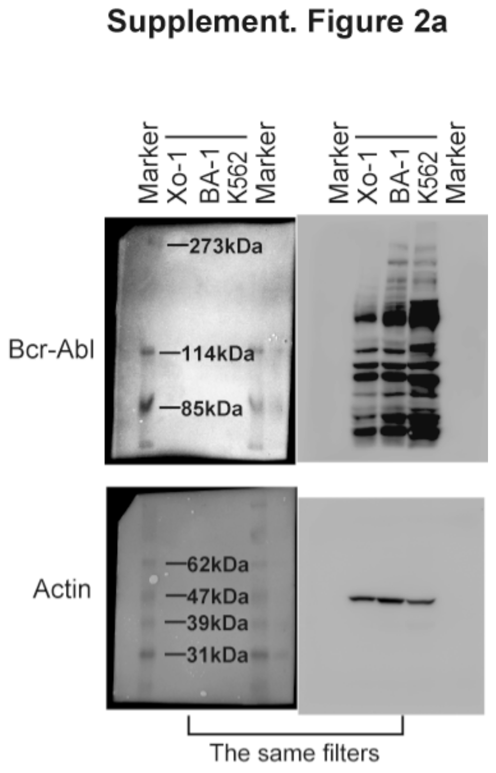

Supplementary Figure 10. Full scan of blots for figure 8a, and supplementary figure 2a. Prestained molecular markers were used.

Supplementary Table 1. Antibodies use for FACS analysis, cell sorting and immunostaining

Antibody	Clone	Company	Catalogue No.	Used concentration
**FACS analysis and Cell sorting**				
anti-CD3-PE	145-2C11	eBioscience	12-0031-85	1ug/ml
anti-CD4-PE	GK1.5	eBioscience	12-0041-85	1ug/ml
anti-CD8-PE	53-6.7	eBioscience	12-0081-85	1ug/ml
anti-B220-PE	RA3-6B2	eBioscience	12-0452-85	1ug/ml
anti-GR1-PE	RB6-8C5	eBioscience	12-5931-85	1ug/ml
anti-CD11b-PE	M1/70	eBioscience	12-0012-85	1ug/ml
anti-TER119-PE	TER-119	Biolegend	116208	1ug/ml
anti-CD140a-PE	APA5	Biolegend	135905	1ug/ml
anti-F4/80-PE	BM8	TONBO	50-4801-U100	1ug/ml
anti-CD45-APC	30-F11	TONBO	20-0451-U100	1ug/ml
anti-TER119-APC-Cy7	TER119	Biolegend	116223	1ug/ml
anti-CD4-APC	GK1.5	Biolegend	100412	1ug/ml
anti-CD4-PE-Cy7	GK1.5	TONBO	60-0041-U100	1ug/ml
anti-CD8a-PE-Cy7	53-6.7	TONBO	60-0081-U101	1ug/ml
anti-CD11b-PE-Cy7	M1/70	TONBO	60-0112-U102	1ug/ml
anti-CD62L-PE-Cy7	MEL-14	TONBO	60-0621-U103	1ug/ml
anti-CD31-APC	390	Biolegend	102410	1ug/ml
anti-CD45R-APC	RA3-6B2	Biolegend	103226	1ug/ml
anti-CD4-APC-Cy7	GK1.5	Biolegend	100414	1ug/ml
anti-CD8a-APC-Cy7	53-6.7	TONBO	25-0081-U100	1ug/ml
anti-CD45-APC-Cy7	30-F11	Biolegend	103116	1ug/ml
anti-Gr-1-APC-Cy7	RB6-8C5	Biolegend	108424	1ug/ml
anti-CD11c-Pacific Blue	N418	Biolegend	117322	1ug/ml
anti-TCRβ-BV421	H57-597	Biolegend	109226	1ug/ml
anti-CD8a-BV510	53-6.7	BD Bioscience	563068	1ug/ml
anti-CD45-BV510	30-F11	BD Bioscience	563891	1ug/ml
anti-TER119-BV510	TER-119	BD Bioscience	563995	1ug/ml
anti-Ccl5-PE(RANTES)	2E9/CCL5	Biolegend	149103	1ug/ml
**Immunostaining**				
anti-CD105	MJ7/18	Biolegend	102401	2.5ug/ml
anti-GFP	Rabbit IgG	Invitrogen	A-11122	2ug/ml
anti-vimentin	Rabbit	CST	5741	x100
anti-CD11c	HL3	BD Bioscience	500283	0.125ug/ml
anti-CD3	145-2C11	Biolegend	100302	2.5ug/ml
anti-B220	RA3-6B2	BioLegend	103202	2.5ug/ml

Supplementary Table 2. Primer sets used for qPCR

Gene name	Forward primer 5'-3'	Reverse primer 5'-3'
Actb	GGCTGTATTCCCCTCCATCG	CCAGTTGGTAACAATGCCATGT
aSMA	CCAGCACCATGAAGATCAAG	TCCACATCTGCTGGAAGGTA
aSMA-n	GTCCCAGACATCAGGGAGTAA	TCGGATACTTCAGCGTCAGGA
Ccl2	CATCCACGTGTTGGCCA	GATCATCTTGCTGGTGAATGAGT
Ccl3	CAAGTCTTCTCAGCGCCATA	GGAATCTTCCGGCTGTAGG
Ccl5	CCTACTCCCACTCGGTCCT	GCTGATTTCTTGGGTTTGCT
Col1a2	CACCTGGTCCTGTTGGAAGT	CACCAGGGAAGCCAGTCA
Col3a1	GCACAGCAGTCCAACGTAGA	GACATCTCTAGACTCATAGGACTGACC
Col6a5	GCAATGGCGAGTCTCACTTC	CTCATGAGCAAGACCCAGTTC
Cxcl1	AGACTCCAGCCACACTCCAA	TGACAGCGCAGCTCATTG
Cxcl5	GGGAAACCATTGTCCCTGA	TCCGATAGTGTGACAGATAGGAAA
Cxcl9	CCATGAAGTCCGCTGTTCTT	TGAGGGATTTGTAGTGGATCG
Cxcl10	ATCAGCACCATGAACCCAAG	TTCCCTATGGCCCTCATTCT
Cxcl11	GCTGCTGAGATGAACAGGAA	TTCCCTATGGCCCTCATTCT
Cxcl12	CCAAACTGTGCCCTTCAGAT	ATTTCGGGTCAATGCACACT
Cxcl13	ATGAGGCTCAGCACAGCA	ATGGGCTTCCAGAATACCG
Fgfr1	TCTGGCCTCTACGCTTGC	GAGGATGGGAGTGCATCTG
Fgf1	CAGCCTGCCAGTTCTTCAG	GGCTGCGAAGGTTGTGAT
Fgf2	CGGCTCTACTGCAAGAACG	TGCTTGGAGTTGTAGTTTGACG
Fgf7	TGGCTGACACCATGACTAGC	GGCTACAGGCTGTCGTTTTT
Fgf10	CGGGACCAAGAATGAAGACT	GCAACAACTCCGATTTCCAC
Itga1	GATGGGGACGTCAACATTCT	TGTGGTTAAGACGCTACCAAAG
Itga5	CACCATTCAATTTGACAGCAA	TCCTCTCCCTTGGCACTGTA
Itga7	CGTGCTCTGGACTCTGTGG	CCCAGCTCACACTCGACAT
Itga8	TGGAGAATTCACTGGGGACT	AAGTTCTGTGCTCCTCTTGGA
Itga9	GACATTGATGATGACGGGTTC	TGTAGACTGCGCCAGCAA
Itga11	GCAGACGTCCTCTTTACCAGA	GAGCTGTTTGCCTTGACCTC
ItgaV	GGTGTGGATCGAGCTGTCTT	CAAGGCCAGCATTTACAGTG
Itgb1	TCAGATCCAACCACAACAGC	GGTAATCTTCAGCCCTCTTGAA
Itgb3	GCCATCATGCAGGCTACAG	AAACACTAGCAAATGGGATGC
Itgb5	ACCTGCCAAGATGGCATATC	CACGGACACTTCAAAGGATG
Pdgfa	GGCTGGCTCGAAGTCAGATC	CCTCAGCCCCTACGGAGTCT
Pdgfra	AAGACCTGGGCAAGAGGAAC	GAACCTGTCTCGATGGCACT
Pdgfrb	TGCAGAGACCTCAAAAGGTG	GAAAGTCACATTCGTTTCTAGCTG
Sca-1	CCTACCCTGATGGAGTCTGTGT	GGCAGATGGGTAAGCAAAGA
Snai1	CTTGTGTCTGCACGACCTGT	CAGGAGAATGGCTTCTCACC
Snai2	ACATTGCCTTGTGTCTGCAA	GAAAGGCTTTTCCCCAGTGT
Vimentin	CCAACCTTTTCTTCCCTGAAC	TTGAGTGGGTGTCAACCAGA
Vcam1	TCTTACCTGTGCGCTGTGAC	ACTGGATCTTCAGGGAATGAGT
